# Metagenomic and metaproteomic analyses of microbial amino acid metabolism during Cantonese soy sauce fermentation

**DOI:** 10.3389/fnut.2023.1271648

**Published:** 2023-11-03

**Authors:** Cong Chen, Lin Feng Wen, Li Xin Yang, Jun Li, Qi Xin Kan, Ting Xu, Zhan Liu, Jiang Yan Fu, Yong Cao

**Affiliations:** ^1^College of Food Science, Guangdong Provincial Key Laboratory of Nutraceuticals and Functional Foods, Guangdong Natural Active Object Engineering Technology Research Center, South China Agricultural University, Guangzhou, China; ^2^Guangdong Eco-Engineering Polytechnic, Guangzhou, China; ^3^Jonjee Hi-Tech Industrial and Commercial Holding Co., Ltd., Zhongshan, China; ^4^Guangdong Meiweixian Flavouring Foods Co., Ltd., Zhongshan, China

**Keywords:** Cantonese soy sauce, fermentation, microbial amino acid metabolism, metagenomic, metaproteomic

## Abstract

Cantonese soy sauce is an important type of traditional Chinese brewed soy sauce that was developed in southern China, mainly in Guangdong. Due to the long fermentation period and complex microbiota in Cantonese soy sauce, there are few reports on the microbial metaproteomics of Cantonese soy sauce. In this study, integrative metagenomic and metaproteomic analyzes were used to identify the changes in the dominant microbiota and amino acid synthesis-related enzymes and metabolism during Cantonese soy sauce fermentation. Metagenomic analysis revealed that *Tetragenococcus halophilus*, *Weissella confusa*, *Weissella paramesenteroides*, *Enterobacter hormaechei*, and *Aspergillus oryzae* were the dominant microbiota. Using the Top 15 dominant microbiota identified by metagenomics as the database, LTQ Orbitrap Velos Pro ETD mass spectrometry was used to obtain metaproteomic information about the microbes in the soy sauce, and the results indicated that the active enzymes involved in the metabolism of amino acids were secreted by microorganisms such as *A. oryzae*, *T. halophilus*, and *Zygosaccharomyces rouxii*. During the Cantonese soy sauce fermentation process. Among them, early fermentation (0-15d) was dominated by *A. oryzae* and *T. halophilus*, mid-term fermentation (60-90d) was dominated by *Z. rouxii*, *A. oryzae*, and *T. halophilus*, and late fermentation (90-120d) was dominated by *A. oryzae*, *Z. rouxii*, and *T. halophilus*. Kyoto Encyclopedia of Genes and Genomes analysis revealed that the main enzymes involved in the metabolism of umami amino acids were aspartate aminotransferase, citrate synthase, aconitase, and isocitrate dehydrogenase, which were produced by *Z. rouxii* and *A. oryzae* during early fermentation (0–15 d) and the middle fermentation stage (60–90 d). This study constructed a regulatory network of enzymes potentially involved in the metabolism of flavor amino acids, which provided a theoretical basis for studying the amino acid metabolism of Cantonese soy sauce.

## 1. Introduction

Cantonese soy sauce is an important type of traditional Chinese brewed soy sauce and was developed in southern China, mainly in Guangdong (1). With whole soybeans and wheat flour as the main raw materials, Cantonese soy sauce is naturally brewed by the traditional fermentation technique, such as steaming, koji making, fermentation, and post-processing. During the fermentation process, the sauce mash is placed in an open jar and subjected to natural fermentation for 3–6 months, which brine and sauce mash are periodically mixed. The type and content of amino acids in soy sauce are an important basis for determining soy sauce quality (2). During the soy sauce fermentation process, the population structure and interaction of microorganisms play a key role in the proportion and content of amino acids in the soy sauce (3, 4).

Most previous studies have focused on the analysis of amino acid composition in soy sauce or on the microbial dominant microbiota related to amino acid metabolism in soy-based fermented foods (5–8), while the mechanism of amino acid metabolism during soy sauce fermentation is rarely reported. With the rapid development of meta-omics technology (9, 10), metagenomics (11), metatranscriptomics (12), and metabolomics (13, 14) have been widely used in the study of fermented foods. Comparatively, metaproteomics is less used in the study of fermented foods but can be used to directly study the nature of microbial functions in specific environments and conditions and to elucidate the complex interactions between the substrate and microbial communities (15). Therefore, this study used metagenomic and metaproteomic analyzes to reveal the microorganisms and the main protease substances responsible for producing flavor amino acids during Cantonese soy sauce fermentation.

## 2. Materials and methods

### 2.1. Materials

Soy sauce were obtained from Guangdong Meiweixian Flavouring Foods Co., Ltd. Three fermenters were randomly selected from 100 fermenters (Zhongshan, China), and three biological repeats were made for each sample. At 0 d, 15 d, 30 d, 45 d, 60 d, 90 d, and 120 d, 5 kg of soy sauce mash and 5 liters of salt water were taken from the fermentation tank as a sample, and three biological repeats were made for each sample.

### 2.2. Experimental methods

#### 2.2.1. Sample preprocessing

50 g of soy sauce mash and 50 mL of salt water were mixed well and filtered with gauze. The filtrate was collected and centrifuged at 3000 × g for 5 min at 4°C, and the supernatant was discarded to obtain a bacterial precipitate, which was washed twice with sterile water.

#### 2.2.2. Determination of total nitrogen content and amino acid nitrogen content in soy sauce samples

According to the Kjeldahl method (16) and the *National Food Safety Standard - Determination of amino acid nitrogen in food* (GB5009.235–2016), the TN content and free amino acid nitrogen content in soy sauce samples at different fermentation stages were determined.

#### 2.2.3. Soy sauce metagenomic sequencing and species abundance analysis

The preprocessed soy sauce samples were sequenced through the DNBSEQ sequencing platform, and in the original sequencing data obtained, reads containing 10% uncertain bases (N bases) and low-quality bases >40% (bases with Q < 20) were excluded, and the assembly software MEGAHIT (17) was used to perform metagenomic *de novo* assembly. Salmon software was used to quantify gene abundance, and Kraken was used to annotate species and calculate species abundance.

#### 2.2.4. Protein extraction and identification in soy sauce samples

According to the improved bacterial extraction method of Bestbio Company (18), an equal volume of protein extraction solution (Bacterial Protein Extraction Kit, Bestbio Company) was added, and a bead mill was used at-20°C for 3 min, with grinding for 30 s and rest intervals of 20 s. The sample was placed on ice for 1 h and mixed every 10 min. The sample was sonicated on ice for 5 min and centrifuged for 15 min at 20,000 × g and 4°C. The supernatant was transferred to a new 1 mL precooled centrifuge tube to obtain a protein extract of the soy sauce sample. A protein sample (50 μg) was first removed and subjected to reduction and alkylation treatment, then digested in 25 mM NH_4_HCO_3_ (protein: trypsin = 1:50) overnight at 37°C. Polypeptide samples obtained after ultrafiltration were analyzed by LC-ESI-MS/MS using a Waters 2,695 nanoLC-LTQ Orbitrap Velos Pro ETD system (LTQ Orbitrap Velos Pro ETD mass spectrometer, Thermo Company, United States; Analytical column: C18 analytical column (50 μm × 2 μm × 15 cm), Thermo Company, USA; Waters 2,695 chromatograph: Waters Company, United States; LC-10A High Performance Liquid Chromatograph: Shimadzu Corporation, Japan). The peptide elution gradient is as follows: 0–10 min, 5% B; 10–100 min, 5–40% B; 100–105 min, 40–95% B; 105–115 min, 95% B; 115–116 min, 95–5% B; and 116–120 min, 5% B., mobile phase A was 0.1% (v/v) formic acid (FA), and mobile phase B was acetonitrile (ACN). The LTQ Orbitrap Velos Pro ETD mass spectrometry conditions were as follows: positive ion mode, electrospray ionization (ESI) source, spray voltage 2.0 kV, collision-induced dissociation (CID) mode (scanning in the range of 350–1800 m/z), and acquisition time 120 min.

#### 2.2.5. Data processing and functional analysis

For the downstream mass spectrometry data obtained from the LTQ Orbitrap Velos Pro ETD assay, the Proteome Discoverer 2.5 software was used for protein peptide matching and database search and identification (MS/MS spectra were searched with the SEQUEST engine against the UniProt database^1^ for the top 15 microorganisms in metagenomic abundance). The library search parameters were as follows: trypsin digestion, the maximum number of missed cleavage sites was 2, the mass deviation of the precursor ion was within 10–5, the mass deviation was 0.6 Da, and the false discovery rate (FDR) was <0.01.

Bioinformatics data for successfully identified proteins were obtained by Gene Ontology (GO) and Kyoto Encyclopedia of Genes and Genomes (KEGG) annotation. For the GO^2^ and KEGG pathway^3^ analyzes, the PartiGene program^4^ was used.

## 3. Results and discussion

### 3.1. Determination of TN content and amino acid nitrogen content in the Cantonese soy sauce fermentation process

**Figure 1:**
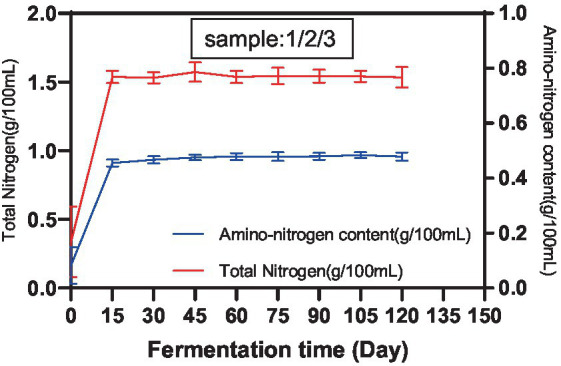
TN content and amino acid nitrogen content of Cantonese soy sauce at different fermentation times.

According to GB5009.235, the Kjeldahl method and the colorimetric method were used to determine the TN content and amino acid nitrogen content of each sample in the Cantonese soy sauce fermentation process, and results are shown in Figure 1. The TN content and amino acid nitrogen content increased rapidly within the first 15 d of fermentation and then remained steady until 120 d, with final concentrations of 1.51 g/100 mL and 0.98 g/100 mL, respectively. The amino acid nitrogen content accounted for 64.90% of the TN content. In the early stage of fermentation, due to sufficient nutrients in the fermenter, the microorganisms multiplied and metabolized rapidly, and the TN content and amino acid nitrogen content increased rapidly. After 15 d of fermentation, limited by the total volumes of the fermenter and soy sauce mash, the overall number of fermenting microorganisms gradually became saturated, and the TN content and amino acid nitrogen content produced by the metabolism of microorganisms stabilized, which is essentially consistent with the results of Bai (19) and Liu (20). However, the ratios of various amino acids were subject to change according to the dominant microbiota involved at different fermentation times and fermentation conditions.

### 3.2. Analysis of the dominant species during the Cantonese soy sauce fermentation process

**Figure 2:**
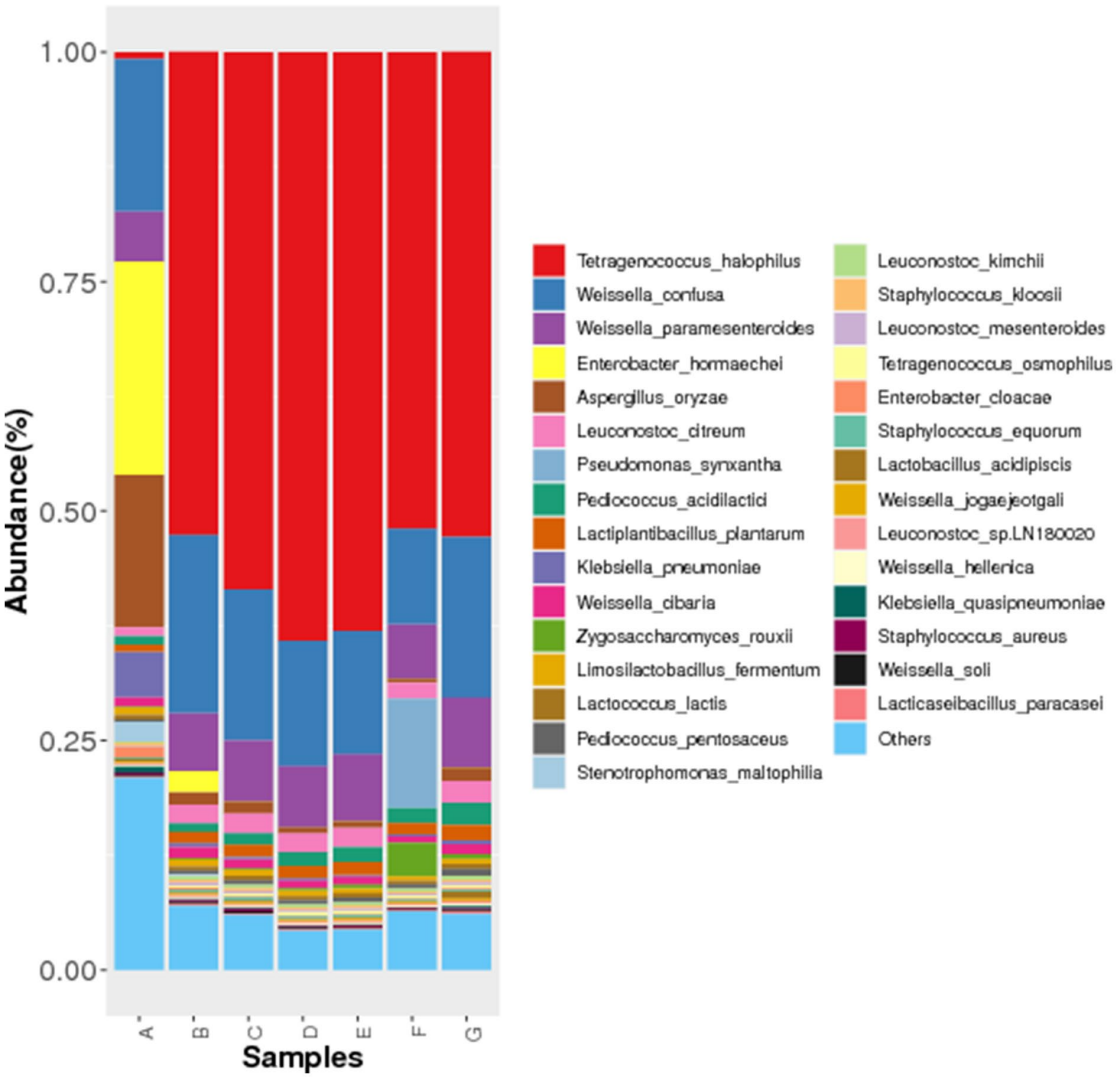
Histogram of microbial species abundance in different Cantonese soy sauce fermentation stages. The abscissa shows the sample name (A-G:0d-120d), and the ordinate shows the relative abundance of the annotated species.

The microorganisms identified in Cantonese soy sauce samples were classified by Kraken, and the results revealed that during the Cantonese soy sauce fermentation process, *Tetragenococcus halophilus*, *Weissella confusa*, *Weissella paramesenteroides*, *Enterobacter hormaechei*, and *Aspergillus oryzae* were the dominant species (Figure 2). This is basically consistent with the main microbiota groups reported in soy sauce in Southeast Asian countries, but the variation patterns and dominant bacterial groups vary from fermentation time to time (21, 22). One of the main reasons could be the different climatic conditions and fermentation processes of soy sauce producing countries. For example, Cantonese soy sauce is a high-salt dilute soy sauce, the concentration of salt water is 18.5 ~ 20.5%, the amount of salt water is 2 ~ 2.5 times of the mash, so sauce mash is floating on the salt water and in contact with the air, which is different from the high salt solid fermentation of Korean soy sauce and the relatively independent closed fermentation of Japanese soy sauce.

In the early stage of fermentation, *W. confusa*, *E. hormaechei*, and *A. oryzae* were the dominant species; after 15 d of fermentation, the abundance of *T. halophilus* increased rapidly; after 30 d of fermentation, the abundance of *E. hormaechei* was reduced rapidly. In the middle stage of fermentation (30–60 d), *Leuconostoc citreum* gradually grew, and *T. halophilus*, *W. confusa*, *W. paramesenteroides*, and *A. oryzae* formed the dominant species; *Pediococcus acidilactici* grew rapidly at 90 d of fermentation and then declined rapidly at 120 d, which is possibly related to the Cantonese soy sauce fermentation process. For example, in the early stage of fermentation (0–15 d), after starter-making using *A. oryzae* and other species is completed, a large amount of salt water and starter are mixed, so *T. halophilus* could replace *W. confusa*, *E. hormaechei*, and *A. oryzae* as the dominant species. During the fermentation process, the mash and salt water are mixed regularly (every 15 or 30 days), which leads to a change in fermentation conditions at different fermentation times, resulting in changes in the dominant species and the production of various secondary metabolites (23, 24). Therefore, the time points where substantial changes occur in these microbial communities were the focus of this research.

### 3.3. Metaproteomic analysis of Cantonese soy sauce during the fermentation process

**Figure 3:**
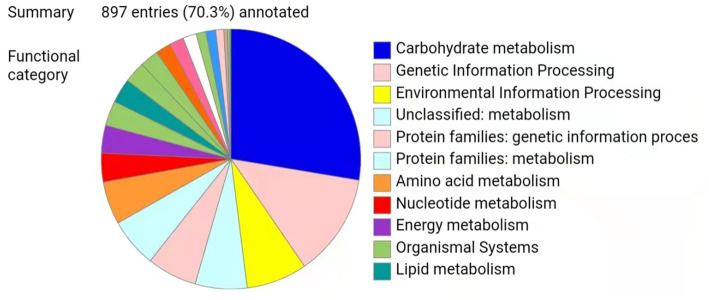
Overall KEGG results.

Metaproteomics can be used to explore the functions and key enzymes of microorganisms in fermented foods at the protein level and to enhance the understanding of fermented food ecosystems (25–27). To date, there have been many proteomic studies on *A. oryzae*, the dominant specie in high-salt liquid-state fermentation soy sauce (HLFSS), and few studies on microbial metaproteomics in HLFSS (28, 29), possibly due to the long fermentation period of Cantonese soy sauce and its diverse microbial populations and complex metabolites, along with the lack of sample preparation methods for microbial proteomics analysis. The improved bacterial protein extraction method of Bestbio Company is a set of proteomic sample preparation methods suitable for soy sauce samples (18). Through the experimental steps such as gauze filtration and centrifugal precipitation, we mainly obtained the microorganisms in the soy sauce fermentation process, and the subsequent Metaproteomic analysis was based on the proteins in the microbial cells. In this study, the top 15 species in the metagenome were used as the comparison database. The pd2.5 software was used to identify 1,082 proteins in 21 samples (three biological replicates for each time point) at seven time points (0 d, 15 d, 30 d, 45 d, 60 d, 90 d, and 120 d) during soy sauce fermentation, and the intergroup differentially expressed proteins (DEPs) were compared to explore the molecular mechanisms of the soy sauce fermentation process. This study found that there are 76 upregulated proteins and 173 downregulated proteins at 30 d of fermentation, 186 upregulated proteins and 77 downregulated proteins at 60 d, 94 upregulated proteins and 189 downregulated proteins at 90 d, and 91 upregulated proteins and 134 downregulated proteins at 120 d. Overall, the differences between 0 d, 30 d, 60 d, and 90 d were obvious and varied. This is consistent with the predominant microbial populations detected by the metagenomic analysis, indicating that changes in microbial populations and enzymes during these periods may be closely related to the formation of the flavor of Cantonese soy sauce. All 1,082 identified proteins were imported into KEGG for analysis, and the annotation results of 897 enzymes were obtained, as shown in Figure 3. Among them, 39 enzymes were identified in the metabolic pathway of flavor amino acids, accounting for 4.35%. It indicates that amino acid metabolism plays a relatively important role in the entire metabolic pathway during the fermentation process of Cantonese soy sauce.

**Figure 4:**
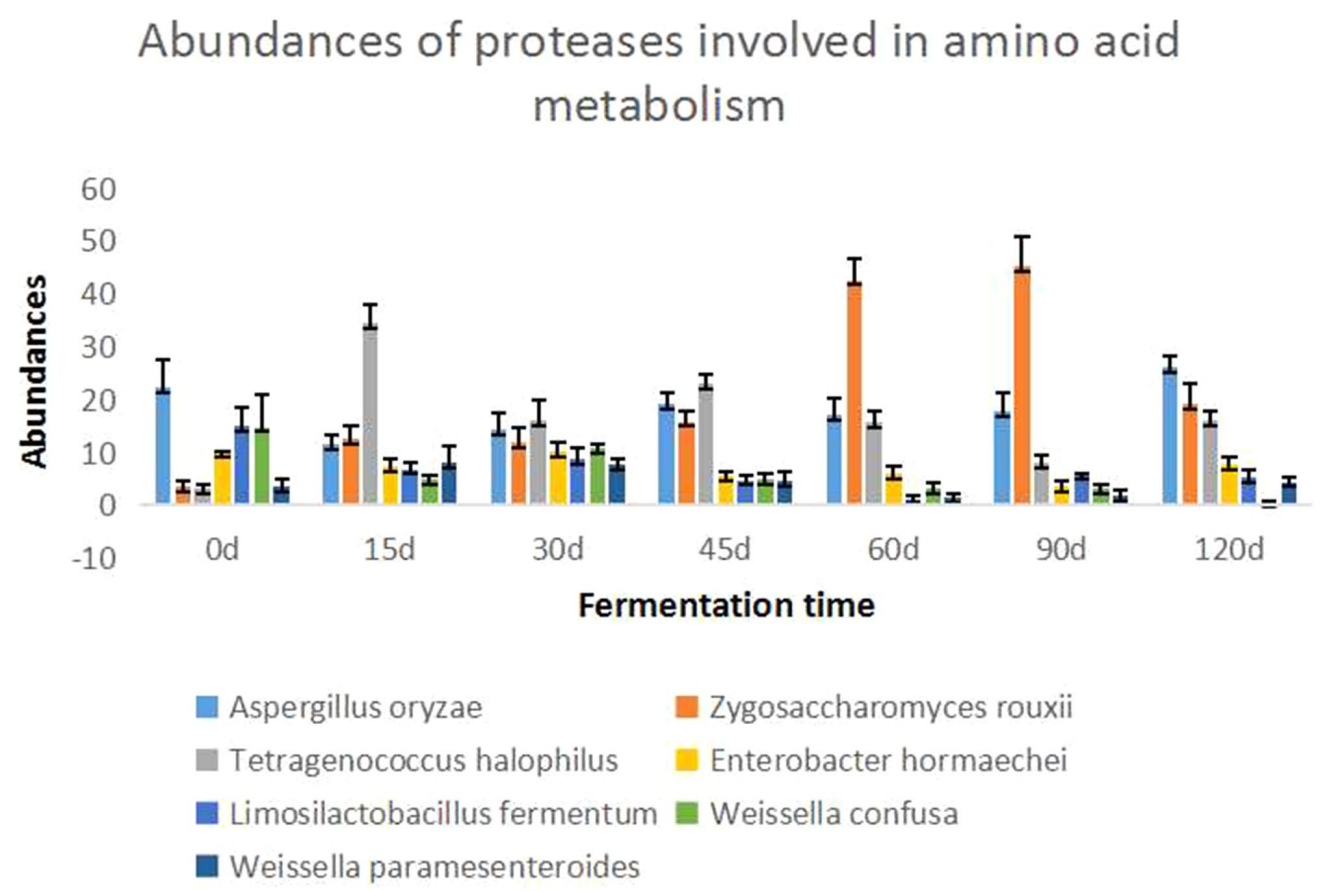
Abundance map of amino acid-metabolizing enzymes in Cantonese soy sauce at different fermentation times. The abscissa shows the fermentation time, and the ordinate shows the relative abundance of the seven dominant species at each fermentation stage.

The seven dominant species with the highest abundance of enzymes involved in amino acid metabolism were selected, and the changes in enzymes involved in amino acid metabolism at different fermentation times during the Cantonese soy sauce fermentation process were compared, as shown in Figure 4. In the early fermentation stage (0–15 d), the protein abundance of *T. halophilus* and *Zygosaccharomyces rouxii* increased rapidly; in the middle fermentation stage (45–60 d), the protein abundance of *Z. rouxii* began to increase; and in the late fermentation stage (90–120 d), the protein abundance of *A. oryzae* and *T. halophilus* began to increase, while that of *Z. rouxii* decreased. The dominant species in different fermented foods or in different production processes of the same fermented foods are different, however, studying the relationship between the metabolism of dominant bacteria and the flavor of fermented food, we can simplify the complexity and quickly find some key enzymes related to flavor. Zhang et al. (30) and Xie et al. (31) reveal microbial assortments and key enzymes in Dajiang by metagenomic and metaproteomic analyzes. Belda et al. (32) proposed an overview of genetic and transcriptional studies to explain and interpret the effects of microorganisms in wine aroma and flavor. In this study, the amino acid metabolism enzymes involved in Cantonese soy sauce fermentation, *A. oryzae* and *Weissella* are dominant on Day 0; *T. halophilus*, *A. oryzae*, and *Z. rouxii* are dominant on Day 15; *Z. rouxii*, *A. oryzae*, and *T. halophilus* are dominant on Day 60; and *A. oryzae*, *Z. rouxii*, and *T. halophilus* are dominant on Day 120.

### 3.4. Analysis of the possible metabolic pathways of flavor amino acids during Cantonese soy sauce fermentation

**Figure 5:**
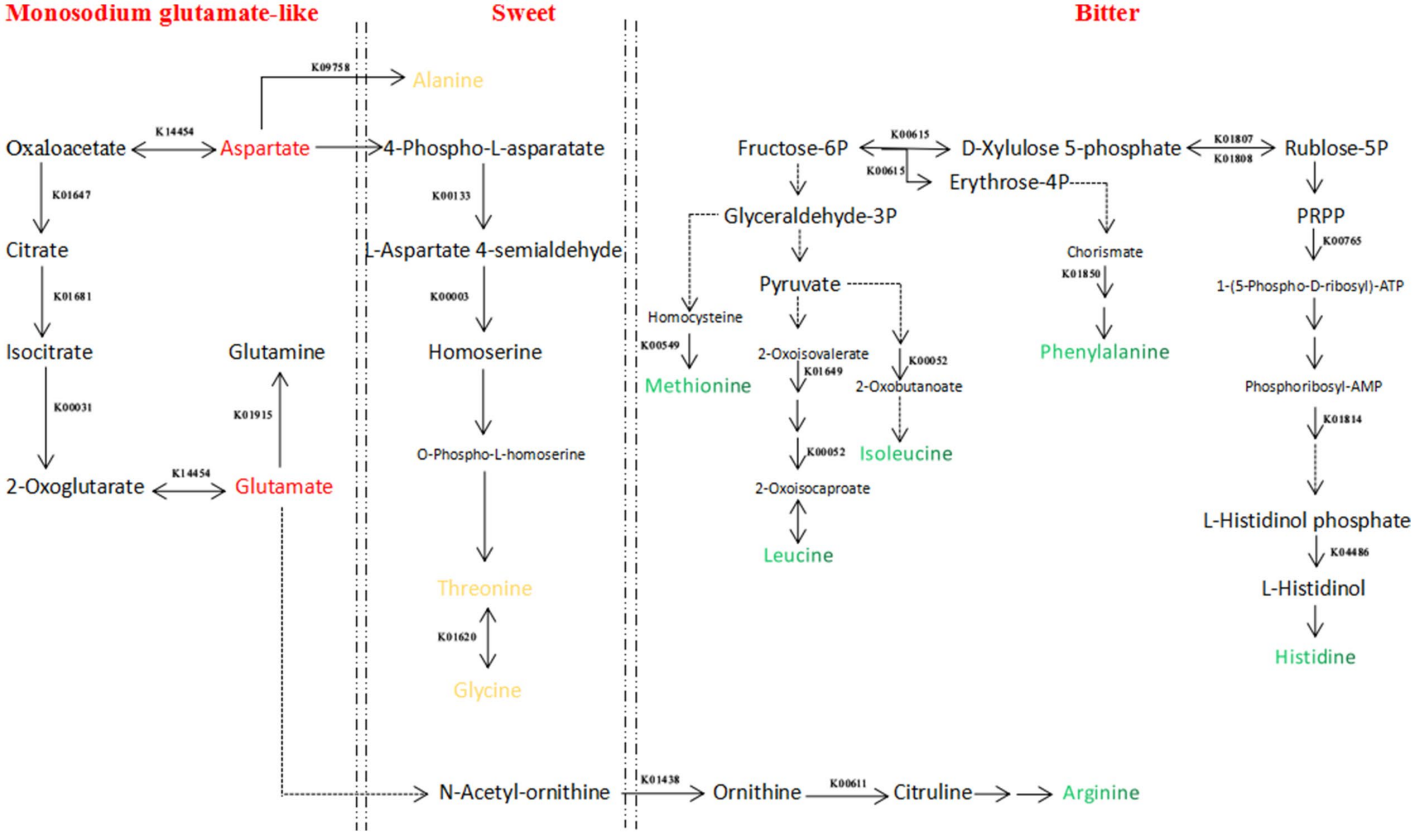
Regulatory network diagram of flavor amino acid metabolism during Cantonese soy sauce fermentation. Red indicates umami amino acids, yellow indicates sweet amino acids, and green indicates bitter amino acids. Solid arrows indicate that the reaction can be completed in one step; dashed arrows indicate that the reaction can be completed in multiple steps.

According to the method of Tseng et al., flavor amino acids are divided into umami, sweet, bitter, and flavorless amino acids, and 39 related enzymes were identified in the metabolic pathways of umami, sweet, and bitter amino acids. The changes in abundance at different fermentation times are shown in Table 1. A regulatory network of these enzymes involved in the metabolism of flavor amino acids was constructed (Figure 5). However, due to the limitations of Label-Free quantitative proteomics, only preliminary inferences can be made based on large data sets to identify possible regulatory networks, and these inferred key enzymes still need further validation.

**Table 1:** Changes in the abundance of enzymes involved in the metabolism of flavor amino acids during Cantonese soy sauce fermentation.

KO No.	KO Definition	Protein accession No.	Protein name and organism	Exp. q-value	Matched Peptides/Coverage/Score	MW [kDa]	calc.pI	Changes in regulation intensity*
Monosodium glutamate-like (Asp Glu)								
K14454	Aspartate aminotransferase	GAV52403.1	Hypothetical protein ZYGR_0AG03940 [*Zygosaccharomyces rouxii*]	0.000	3/15/19	46.3	6.99	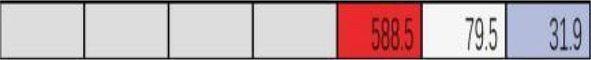
K01647	Citrate synthase	CAR30866.1	ZYRO0E04290p [*Zygosaccharomyces rouxii*]	0.000	3/8/33	52.4	8.44	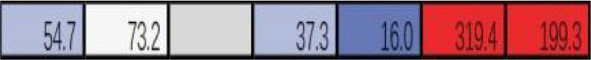
K01681	Aconitate hydratase	CAR30007.1	ZYRO0G22154p [*Zygosaccharomyces rouxii*]	0.000	8/12/66	85.2	6.71	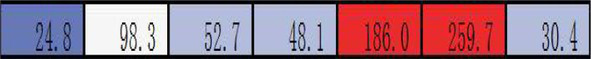
		GAV56395.1	Hypothetical protein ZYGR_0BB01730 [*Zygosaccharomyces rouxii*]	0.000	8/1466	85	6.61	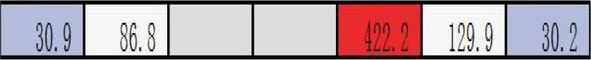
		EIT76572.1	Aconitase/homoaconitase [*Aspergillus oryzae* 3.042]	0.004	1/2/3	85.5	6.93	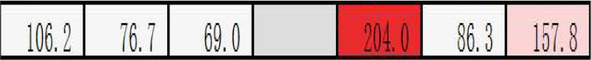
K00031	Isocitrate dehydrogenase	OOO12471.1	Isocitrate dehydrogenase, NADP-dependent [*Aspergillus oryzae*]	0.000	3/5/38	80.7	8.79	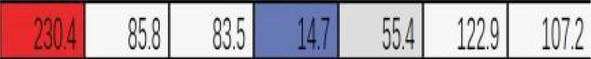
K01915	Glutamine synthetase	VAF75852.1	Glutamine synthetase [*Enterobacter hormaechei*]	0.004	1/5/10	51.8	5.53	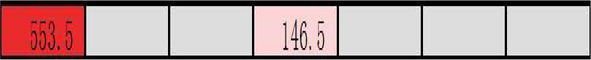
Sweet (Ala Gly Thr)								
K01620	Threonine aldolase	BAE55552.1	Unnamed protein product [*Aspergillus oryzae* RIB40]	0.000	4/16/28	40	6.14	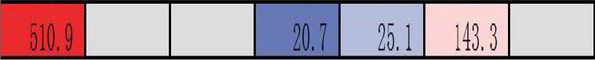
K09758	Aspartate 4-decarboxylase	GFK25291.1	Aspartate aminotransferase [*Tetragenococcus halophilus*]	0.004	1/2/ 3	59.8	5	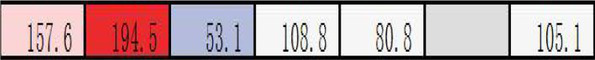
K00133	Aspartate-semialdehyde dehydrogenase	GAV50367.1	Hypothetical protein ZYGR_0U02230 [*Zygosaccharomyces rouxii*]	0.000	3/11/5	39.2	6.62	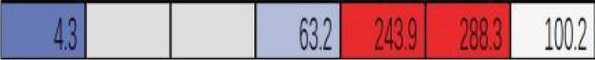
		QBZ05143.1	Aspartate-semialdehyde dehydrogenase [*Weissella confusa*]	0.004	1/9/4	39.4	4.82	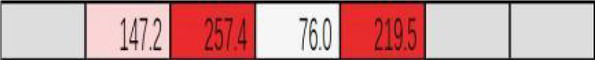
K00003	Homoserine dehydrogenase	EIT76589.1	Homoserine dehydrogenase [*Aspergillus oryzae* 3.042]	0.003	1/5/14	42.6	8.53	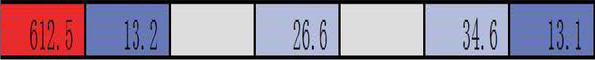
Bitter (Arg His Ile Leu Met Phe Try Val)								
K01438	argE; acetylornithine deacetylase	XP_001819296.1	Unnamed protein product [*Aspergillus oryzae* RIB40]	0.000	3/16/45	45.7	5.01	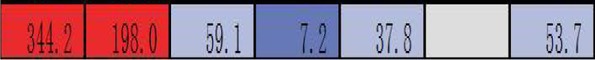
K00611	OTC, argF, argI; ornithine carbamoyltransferase	QQC61473.1	Ornithine carbamoyltransferase [*Pediococcus pentosaceus*]	0.000	5/2692	37.1	5.2	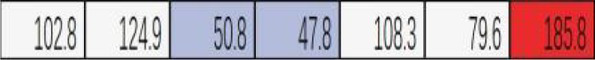
		GFK23200.1	Ornithine/putrescine carbamoyltransferase [*Tetragenococcus halophilus*]	0.000	4/17/70	38	5.1	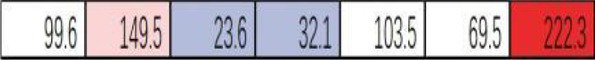
		GFK21365.1	Ornithine/putrescine carbamoyltransferase [*Tetragenococcus halophilus*]	0.000	4/20/55	37.2	5.14	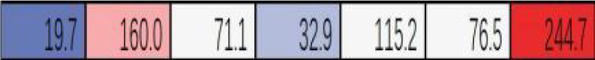
		WP_004905047.1	Ornithine carbamoyltransferase [Leuconostoc]	0.000	3/12/47	38.9	5.76	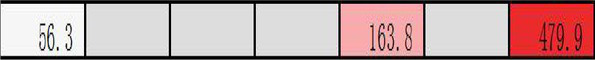
		GEO56406.1	Ornithine carbamoyltransferase, catabolic [*Weissella confusa*]	0.000	2/9/17	38.9	5.3	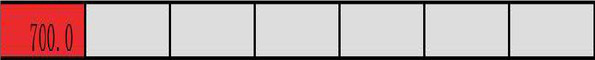
		EIT78478.1	Ornithine carbamoyltransferase OTC/ARG3 [*Aspergillus oryzae* 3.042]	0.003	1/4/2	40.4	8.16	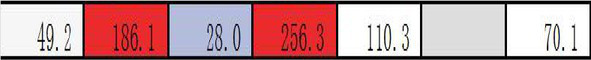
K00765	hisG; ATP phosphoribosyltransferase	EIT73892.1	ATP phosphoribosyltransferase [*Aspergillus oryzae* 3.042]	0.000	6/24/40	32.5	5.33	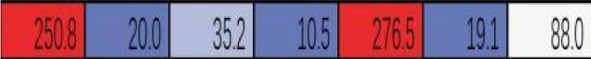
K01814	hisA; phosphoribosyl formimino-5-aminoimidazole carboxamide ribotide isomerase	GAV54177.1	Hypothetical protein ZYGR_0AK06790 [*Zygosaccharomyces rouxii*]	0.000	1/8/19	28.6	6.02	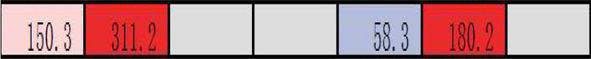
K04486	Histidinol-phosphatase (PHP family)	GFK28292.1	Histidinol phosphatase [*Tetragenococcus halophilus*]	0.000	6/33/159	30.3	4.96	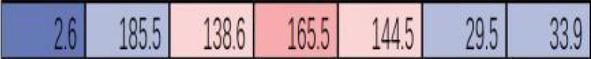
K00052	leuB, IMDH; 3-isopropylmalate dehydrogenase	CBC92325.1	Unnamed protein product [*Aspergillus oryzae*]	0.000	4/15/38	38.6	5.45	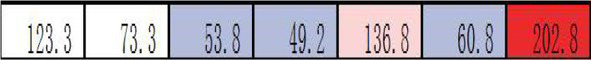
K01649	leuA, IMS; 2-isopropylmalate synthase	BAE56178.1	Unnamed protein product [*Aspergillus oryzae* RIB40]	0.000	1/3/10	71.1	5.74	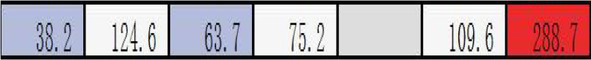
K07173	luxS; S-ribosylhomocysteine lyase	RZQ57604.1	Ribosylhomocysteine lyase [*Weissella paramesenteroides*]	0.000	1/9/28	18	5.53	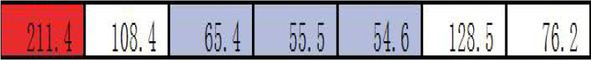
		GFK23347.1	S-ribosylhomocysteinase [*Tetragenococcus halophilus*]	0.000	1/9/7	17.7	5.14	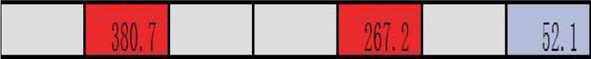
		QSH37704.1	S-ribosylhomocysteine lyase [*Limosilactobacillus fermentum*]	0.003	1/9/4	17.7	5.52	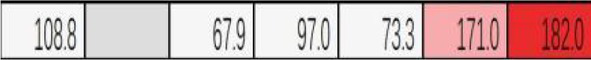
K00549	metE; 5-methyltetrahydropteroyltriglutamate--homocysteine methyltransferase	OOO14364.1	Methyltetrahydropteroyltriglutamate--homocysteine methyltransferase [*Aspergillus oryzae*]	0.000	6/14/91	87.1	6.95	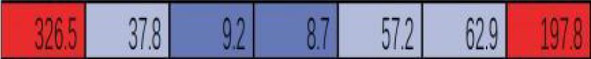
		CAR26154.1	ZYRO0B03036p [*Zygosaccharomyces rouxii*]	0.000	3/5/39	86.1	6.42	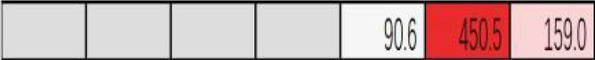
		QSH37705.1	5-Methyltetrahydropteroyltriglutamate--homocysteine S-methyltransferase [*Limosilactobacillus fermentum*]	0.004	1/4/3	41.3	4.87	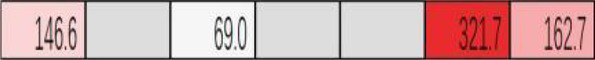
K00789	metK, MAT; S-adenosylmethionine synthetase	STR38571.1	Adenosylmethionine synthetase [*Klebsiella pneumoniae*]	0.000	1/6/57	41.9	5.26	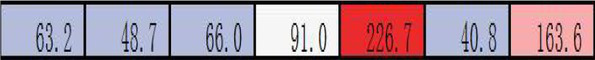
		OOO09136.1	Adenosylmethionine synthase [*Aspergillus oryzae*]	0.002	1/4/4	42.5	5.88	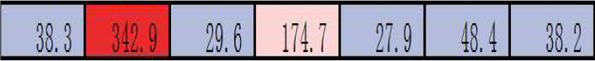
K01850	Chorismate mutase	KDE75461.1	Chorismate mutase [*Aspergillus oryzae* 100–8]	0.004	1/6/3	30.5	5.38	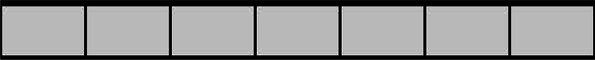
K00615	Transketolase	GFK28833.1	Transketolase [*Tetragenococcus halophilus*]	0.000	10/29/153	72.2	4.89	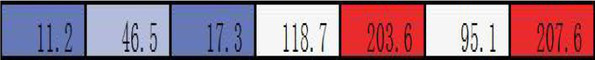
		OOO11256.1	Transketolase [*Aspergillus oryzae*]	0.000	4/9/24	74.8	6.4	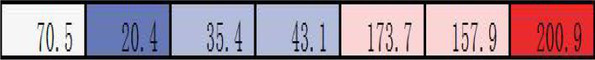
K01807	Ribose 5-phosphate isomerase A	GFK20655.1	Ribose-5-phosphate isomerase A [*Tetragenococcus halophilus*]	0.000	2/16/49	24.6	5.54	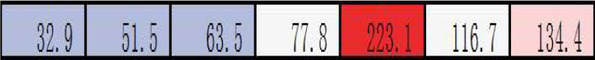
		KDE81361.1	Ribose 5-phosphate isomerase [*Aspergillus oryzae* 100–8]	0.000	1/7/12	34.9	8.84	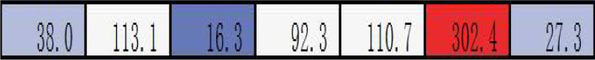
K01808	Ribose 5-phosphate isomerase B [EC:5.3.1.6]	GFK23787.1	Galactose-6-phosphate isomerase subunit LacB [*Tetragenococcus halophilus*]	0.000	1/9/15	17.1	5.58	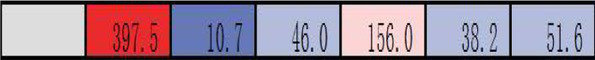
		OOO14769.1	Ribose/galactose isomerase [*Aspergillus oryzae*]	0.009	1/7/6	17.2	7.33	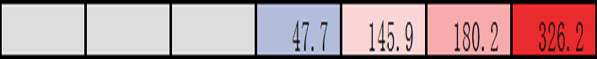

*Changes in regulation intensity: seven small squares represent the protein expression abundance of soy sauce samples at 0 d, 15 d, 30 d, 45 d, 60 d, 90 d, and 120 d from left to right. The color of the squares represents the abundance, with red having a greater abundance than blue, which has a greater abundance than colorless. The deeper the color, the greater the abundance.

Four enzymes were identified in the umami amino acid metabolism pathway: K14454 (aspartate aminotransferase (AST)), K01647 (citrate synthase (CS)), K01681 (aconitase (ACO)), and K00031 (isocitrate dehydrogenase (IDH)). K14454 (AST) is the key enzyme, derived from *Z. rouxii*, and is a reversible enzyme that mediates the conversion of α-ketoglutarate to glutamic acid and oxaloacetate to aspartic acid. Glutamic acid can produce gamma-aminobutyric acid (GABA) under the action of glutamic acid decarboxylase, which could enhance the functionality ofsoy sauce (33, 34) and the abundance of aspartic acid provides the material basis for the synthesis of alanine, threonine, and glycine (35). In terms of changes, the abundance of K14454 was the greatest on Day 60 of the Cantonese soy sauce fermentation process and then decreased rapidly. However, K14454 was largely undetected at 0–45 d, probably because the environment in the early stage of fermentation was not suitable for *Z. rouxii* growth; this result is in general agreement with the changing abundance of *Z. rouxii* detected by the metagenomic analysis, indicating that the period from 60 d to 90 d is the best time for *Z. rouxii* proliferation, and K14454 expression is the highest, thus leading to the accumulation of a large amount of umami amino acids. Therefore, we speculate that during Cantonese soy sauce fermentation, among the metabolic pathways related to the synthesis of umami amino acids, *Z. rouxii* and *A. oryzae* contribute the most, and in the period from 60 d to 90 d of fermentation, the abundance of the relevant enzymes reaches its maximum.

Five enzymes were identified in the metabolic pathways of three sweet amino acids. Under the action of K09758 (aspartate 4-decarboxylase (ASD)), aspartic acid is converted into alanine. K09758 (ASD) is secreted by *T. halophilus*, with the greatest abundance at the early fermentation stage (0 d, 15 d), and is continuously expressed at 45 d, 60 d, and 120 d. In the process of synthesizing threonine *via* homoserine with aspartic acid as the starting point, K00133 and K0003 are secreted by *Z. rouxii*, *W. confusa*, and *A. oryzae*; the abundance of *Z. rouxii* at 60 d and 90 d, *W. confusa* at 30 d and 60 d, and *A. oryzae* at 0 d is the highest. Taking threonine as the starting point, in the process of synthesizing glycine under the action of K01620, K01620 is produced by *A. oryzae*, and the expression level is the highest at 0 d and 90 d. Liu et al. (36) added *T. halophilus* and *Z. rouxii* to soy sauce mash during HLFSS fermentation to increase the umami and sweet amino acids by 34.0 and 27.0%, respectively. Studying the molecular mechanism of the production of flavor amino acids in the Cantonese soy sauce fermentation process can provide a theoretical basis for artificially regulating this process.

In conclusion, the enzymes related to the synthesis of umami and sweet amino acids were mainly produced by *Z. rouxii*, *A. oryzae*, *T. halophilus*, and *W. confusa*, and the expression levels were highest during early fermentation (0–15 d) and the middle fermentation stage (60–90 d). K14454 is the key enzyme that is closely related to the synthesis of glutamic acid and aspartic acid, with the highest expression at 60 d, and large quantities of synthesized aspartic acid provide the material basis for the synthesis of sweet amino acids such as alanine, threonine, and glycine.

The flavor of amino acids is closely related to the structure of the side chains. Most amino acids with strong hydrophobicity have a bitter taste (37). In this study, a total of 21 enzymes were detected in the anabolic pathways of five bitter amino acids, i.e., methionine, leucine, isoleucine, arginine, and histidine. K00549 (5-methyltetrahydropteroyltriglutamate--homocysteine methyltransferase) was detected in the methionine synthesis pathway and is synthesized by *A. oryzae*, *Z. rouxii*, and *Limosilactobacillus fermentum*. Whereas *A. oryzae* was abundantly expressed at 0 d and 120 d during fermentation, the other two bacteria were highly expressed at 90 d and 120 d of fermentation. K01649 (2-isopropylmalate synthase) and K00052 (3-isopropylmalate dehydrogenase) were detected in the leucine and isoleucine synthesis pathways, and both were produced by *A. oryzae*, with the highest abundance at Day 120. K01438 (acetylornithine deacetylase) and K00611 (ornithine carbamoyltransferase) were detected in the arginine synthesis pathway, and K01438 (acetylornithine deacetylase) was produced by *A. oryzae*, with the highest expression at 0 d and 15 d. The bacteria that can produce K00611 (ornithine carbamoyltransferase) were *Pediococcus pentosaceus*, *T. halophilus*, *Leuconostoc*, *W. confusa*, and *A. oryzae* 3.042; the abundance of the first three was the highest at 120 d, and the expression levels of the latter two were greatest during the early fermentation stage (0 d-45 d). K00615 (transketolase), K01807 (ribose 5-phosphate isomerase A), K01808 (ribose 5-phosphate isomerase B), K00765 (ATP phosphoribosyltransferase), K01814 (phosphoribosyl formimino-5-aminoimidazole carboxamide ribotide isomerase), and K04486 (histidinol-phosphatase) were detected in the histidine synthesis pathway. K00615 (transketolase) is produced by *A. oryzae* and *T. halophilus*, and the expression was relatively high in the middle and late fermentation stages (60–120 d), with the peak expression at 120 d. K01807 and K01808 were produced by *A. oryzae* and *T. halophilus*, and the expression levels were relatively high in the middle and late fermentation stages (60–120 d). K00765 was produced by *A. oryzae*, with the highest expression at 0 d and 60 d. K01814 was produced by *Z. rouxii*, with the highest expression at 15 d and 90 d. K04486 was produced by *T. halophilus*, with the highest expression at 30 d, 45 d and 60 d.

From the perspective of bacterial species, the enzyme-producing species related to bitterness were *A. oryzae*, *Z. rouxii*, *T. halophilus*, *W. confusa*, *P. pentosaceus*, and *Leuconostoc*. Controlled *P. pentosaceus* and *Leuconostoc* growth during the late fermentation stage (120 d) could provide a basis for reducing the amount of synthesized arginine. On the other hand, from the perspective of fermentation time, the relative abundance of enzymes related to the synthesis of bitter amino acids reached the maximum mainly during the late fermentation stage (90 d, 120 d); therefore, to control the content of bitter amino acids in Cantonese soy sauce, fermentation time optimization could be a suitable meth.

## 4. Conclusion

The Cantonese soy sauce fermentation process is complex. During large-scale production, the type and metabolism of various microorganisms involved in fermentation vary greatly. In this study, metagenomic and metaproteomic methods were used to explore the metabolic mechanism of flavor amino acids in industrially produced Cantonese soy sauce for the first time. The metaproteomic analysis of dominant species identified a total of 39 enzymes related to the metabolism of flavor amino acids during the Cantonese soy sauce fermentation process; the changes in abundance at different fermentation times were analyzed, and a regulatory network for the metabolism of flavor amino acids was constructed. Among the enzymes related to the synthesis of umami and sweet amino acids, K14454 is the key enzyme; it is closely related to the synthesis of sweet amino acids, such as glutamic acid, alanine, threonine, and glycine, and is produced by *Z. rouxii*, *A. oryzae*, *T. halophilus*, and *W. confusa*, with the highest expression levels during early fermentation (0–15 d) and the middle fermentation stage (60–90 d). Twenty-one enzymes related to the synthesis of bitter amino acids were identified, which are mainly produced in the later fermentation stage. The results of this study lay a theoretical foundation for the regulation of flavor amino acids in the large-scale production of Cantonese soy sauce. In future studies, the molecular mechanisms should also be further investigated using multi-omics approaches.

## Data availability statement

The data presented in the study are deposited in the iProX repository, accession number IPX0006854000.

## Author contributions

CC: Data curation, Formal analysis, Investigation, Methodology, Writing – original draft, Writing – review & editing. LW: Formal analysis, Writing – original draft. LY: Software, Writing – original draft. JL: Writing – original draft. QK: Writing – original draft. TX: Writing – original draft. ZL: Writing – review & editing. JF: Writing – review & editing. YC: Conceptualization, Writing – review & editing.

## Footnotes

^1^ http://www.uniprot.org

^2^ http://www.geneontology.org

^3^ http://www.genome.jp/kegg/pathway.html

^4^ http://www.nematodes.org/bioinformatics/annot8r/index.shtml
